# Fatty Liver Index and Lipid Accumulation Product Can Predict Metabolic Syndrome in Subjects without Fatty Liver Disease

**DOI:** 10.1155/2017/9279836

**Published:** 2017-01-17

**Authors:** Yuan-Lung Cheng, Yuan-Jen Wang, Keng-Hsin Lan, Teh-Ia Huo, Yi-Hsiang Huang, Chien-Wei Su, Wei-Yao Hsieh, Ming-Chih Hou, Han-Chieh Lin, Fa-Yauh Lee, Jaw-Ching Wu, Shou-Dong Lee

**Affiliations:** ^1^Taipei Municipal Gan-Dau Hospital, Taipei, Taiwan; ^2^Faculty of Medicine, School of Medicine, National Yang-Ming University, Taipei, Taiwan; ^3^Healthcare Center, Taipei Veterans General Hospital, Taipei, Taiwan; ^4^Division of Gastroenterology and Hepatology, Department of Medicine, Taipei Veterans General Hospital, Taipei, Taiwan; ^5^Department and Institute of Pharmacology, National Yang-Ming University, Taipei, Taiwan; ^6^Institute of Clinical Medicine, School of Medicine, National Yang-Ming University, Taipei, Taiwan; ^7^Division of Translational Research, Department of Medical Research, Taipei Veterans General Hospital, Taipei, Taiwan; ^8^Division of Gastroenterology, Department of Medicine, Cheng Hsin General Hospital, Taipei, Taiwan

## Abstract

*Background*. Fatty liver index (FLI) and lipid accumulation product (LAP) are indexes originally designed to assess the risk of fatty liver and cardiovascular disease, respectively. Both indexes have been proven to be reliable markers of subsequent metabolic syndrome; however, their ability to predict metabolic syndrome in subjects without fatty liver disease has not been clarified.* Methods*. We enrolled consecutive subjects who received health check-up services at Taipei Veterans General Hospital from 2002 to 2009. Fatty liver disease was diagnosed by abdominal ultrasonography. The ability of the FLI and LAP to predict metabolic syndrome was assessed by analyzing the area under the receiver operating characteristic (AUROC) curve.* Results*. Male sex was strongly associated with metabolic syndrome, and the LAP and FLI were better than other variables to predict metabolic syndrome among the 29,797 subjects. Both indexes were also better than other variables to detect metabolic syndrome in subjects without fatty liver disease (AUROC: 0.871 and 0.879, resp.), and the predictive power was greater among women.* Conclusion*. Metabolic syndrome increases the cardiovascular disease risk. The FLI and LAP could be used to recognize the syndrome in both subjects with and without fatty liver disease who require lifestyle modifications and counseling.

## 1. Introduction

Metabolic syndrome comprises risk factors of cardiovascular disease and type 2 diabetes mellitus (DM), including central obesity, dyslipidemia, and high blood pressure and fasting glucose [1]. Nonalcoholic fatty liver disease (NAFLD) used to be considered an incidental pathologic finding in type 2 DM and obesity but was found to be strongly associated with features of subsequent metabolic syndrome and was even included in the definition of metabolic syndrome [2, 3].

Using data from the general population of northern Italy, the fatty liver index (FLI), an algorithm based on triglyceride (TG) concentration, gamma-glutamyl transferase (GGT) level, body mass index (BMI), and waist circumference (WC), was developed to predict the risk of fatty liver disease in the general population [4]. The FLI has been validated by several studies and has been proven to have a strong association with hypertension and type 2 DM [5–9]. As cardiovascular disease, NAFLD, and metabolic syndrome are closely related, the FLI was also found to have a strong association with metabolic syndrome [10, 11].

The lipid accumulation product (LAP) is an index based on two components, WC and TG concentration, and was designed to indicate the risk of cardiovascular disease. As the LAP shares two of the five components of metabolic syndrome, it has been found to be a reliable tool to detect metabolic syndrome as well [12–14].

However, the ability of the FLI and LAP to predict metabolic syndrome in subjects without fatty liver disease, a group of people with cardiovascular risk as well, has not been clarified. Therefore, the present study aimed to determine the association between the two indexes and metabolic syndrome and to further explore their ability to predict metabolic syndrome in subjects without fatty liver disease in a large-scale cohort in Taiwan.

## 2. Materials and Methods

### 2.1. Study Population

**Figure 1:**
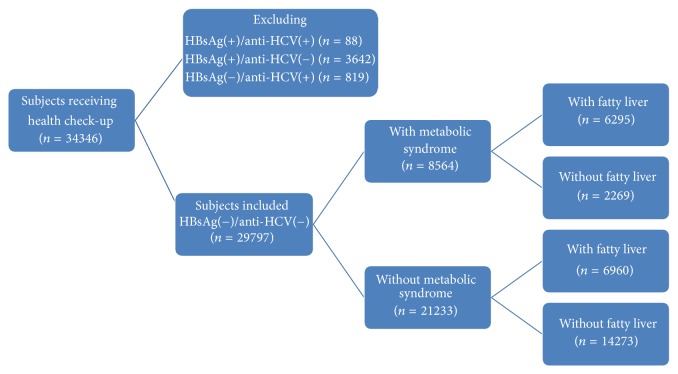
The algorithm for patient selection.

In total, 34,346 subjects received health check-up services provided by internists in the Healthcare Center without hospitalization at the Taipei Veterans General Hospital from 2002 to 2009 [15–18]. Those with hepatitis B virus (HBV) infection, hepatitis C virus (HCV) infection, or HBV/HCV dual infections were excluded, and the remaining subjects were analyzed (Figure 1). All the subjects underwent complete clinical evaluations, laboratory examinations, and abdominal ultrasonography. The BMI was calculated as body weight (in kilograms) divided by the square of body height (in meters). Blood pressure (BP) was measured after the subjects had been seated for more than 5 minutes. The means of three consecutive readings were recorded as the systolic and diastolic BP with a difference in systolic BP < 10 mmHg. A diagnosis of metabolic syndrome was made when three of the following five abnormal findings were met according to the joint interim statement of the International Diabetes Federation Task Force on Epidemiology and Prevention [1]: elevated waist circumference (WC, men ≥ 90 cm or women ≥ 80 cm); TG ≥ 150 mg/dL; low high-density lipoprotein cholesterol (men < 40 mg/dL or women < 50 mg/dL); systolic BP ≥ 130 mmHg and/or diastolic BP ≥ 85 mmHg; and fasting glucose ≥ 100 mg/dL. Impaired fasting glucose (IFG) was defined as an elevated fasting plasma glucose concentration between 100 and 126 mg/dL [19]. Normal or lean subjects were defined as those with a BMI < 23 kg/m^2^ and overweight and obese subjects were defined as those with a BMI ≥ 23 kg/m^2^ [20]. Ultrasonography with Aloka SSD 4000 and 5000 and Philips HD15 was used to diagnose fatty liver disease according to the practice guideline of the American Gastroenterological Association [21]. The FLI was calculated using the following formula: FLI = (*e* 0.953 *∗* loge (TG) + 0.139 *∗* BMI + 0.718 *∗* loge (GGT) + 0.053 *∗* WC − 15.745)/(1 +* e* 0.953 *∗* loge (TG) + 0.139 *∗* BMI + 0.718 *∗* loge (GGT) + 0.053 *∗* WC − 15.745) *∗* 100 [4]. The LAP was calculated using the following formula: LAP = (waist circumference (cm) − 65) × triglycerides (mmol/L) for men and LAP = (waist circumference (cm) − 58) × triglycerides (mmol/L) for women [12].

This study followed the standards of the Declaration of Helsinki and was approved by the Institutional Review Board of Taipei Veterans General Hospital.

### 2.2. Biochemical and Serological Markers

Venous blood samples were collected after an overnight fast. Serum HBV surface antigen was tested by radioimmunoassay (Abbott Laboratories, North Chicago, IL, USA), and HCV antibodies were tested by a second-generation enzyme immunoassay. The serum biochemical markers were measured with a Roche/Hitachi Modular Analytics System (Roche Diagnostics GmbH, Mannheim, Germany).

### 2.3. Statistical Analysis

The study cohort was first divided by metabolic syndrome, and subjects without ultrasonographic fatty liver disease were selected for further analysis. Pearson's chi-squared test and Student's* t*-test were performed to compare categorical and continuous variables with two samples, respectively. Variables with statistical significance (*P* < 0.05) or proximate to it (*P* < 0.1) in univariate analysis were further included in the multivariate analysis using a logistic regression model with the forward stepwise selection procedure. The ability of serum markers to detect ultrasonographic fatty liver disease was examined using the area under the receiver operator characteristic (AUROC) curves. A *P* value less than 0.05 was considered to be statistically significant. All statistical analyses were performed using SPSS 17.0 for Windows (SPSS Inc., Chicago, IL, USA).

## 3. Results

### 3.1. Subject Characteristics Stratified by Metabolic Syndrome

The demographic data of all subjects are summarized in Table 1. The mean age of the population was 52.2 years and 54% was male. Metabolic syndrome was diagnosed in 28.7% of the population. Subjects with metabolic syndrome tended to be older in age, be male, have a higher BMI, serum total cholesterol, low-density lipoprotein cholesterol (LDL-c), alanine aminotransferase (ALT), aspartate aminotransferase (AST), GGT, and fatty liver prevalence, and have lower platelet counts. The FLI averages of subjects with and without metabolic syndrome were 50.2 and 18.0, respectively, while the LAP averages in subjects with and without metabolic syndrome were 64.2 and 23.6, respectively.

We performed a correlation test between total cholesterol and LDL and between ALT and AST. The result showed that the correlation between the sets of data was very high (*r*^2^ = 0.841 and 0.659, resp.). As the collinearity could affect the calculation of individual predictors even though the whole bundle of predictors could still predict the outcome well, we included only total cholesterol and ALT but not LDL and AST in the multivariate analysis to avoid the condition.

**Figure 2:**
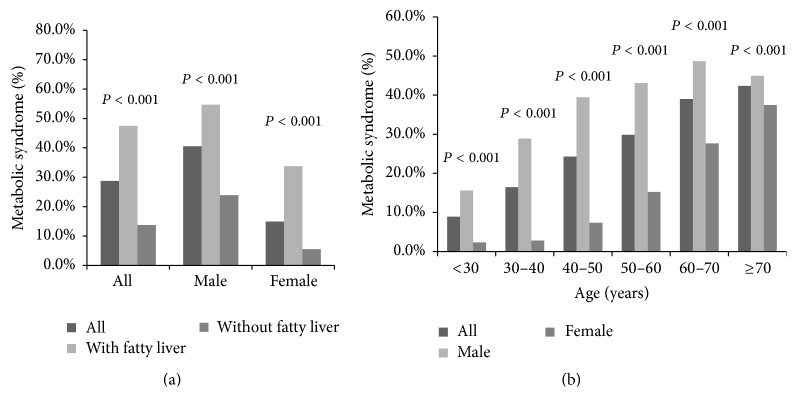
(a) The prevalence of metabolic syndrome divided by the status of fatty liver. (b) The prevalence of metabolic syndrome stratified by age and gender.

By multivariate analysis, ultrasonographic fatty liver disease and male sex were strongly associated with metabolic syndrome (odds ratio: 2.499 and 3.005, resp.), while higher BMI, older age, and higher ALT and GGT were also associated with metabolic syndrome (Table 2). After subjects were divided by ultrasonographic fatty liver disease, the presence of ultrasonographic fatty liver disease was strongly associated with metabolic syndrome as shown in Figure 2(a). We further stratified subjects by age and sex, and the result revealed that male subjects had higher prevalence of metabolic syndrome, and the prevalence of metabolic syndrome increased with age (Figure 2(b)).

### 3.2. Validation of the FLI and LAP for Identifying Metabolic Syndrome

The discriminative ability of the FLI and LAP to identify metabolic syndrome was determined by comparing their AUROC values. The AUROC curve values of the LAP and FLI for the prediction of metabolic syndrome were 0.884 and 0.875, respectively (Table 3). After the subjects were stratified by sex, the AUROC curve values of the LAP and FLI were 0.927 and 0.916, respectively, in women and 0.856 and 0.818, respectively, in men. These values were higher than those of other variables such as BMI, fasting glucose, ALT, GGT, and TG to predict the presence of the metabolic syndrome.

### 3.3. Subject Characteristics Stratified by the IFG and Validation of the FLI and LAP for Identifying IFG

IFG was diagnosed in 21.8% of the population. Subjects with IFG had similar characteristics to those with metabolic syndrome. They tended to be older in age, be male, have a higher BMI, ALT, GGT, and fatty liver prevalence, and have lower platelet counts. The FLI averages of subjects with and without IFG were 38.15 and 23.3, respectively, while the LAP averages were 46.51 and 30.69, respectively (Table 4). The discriminative ability of FLI and LAP to identify IFG was better in subjects without fatty liver disease (0.669 and 0.643, respectively) than in subjects with fatty liver disease. The FLI and LAP also predicted IFG in subjects with lower BMI (0.673 and 0.642, resp.) (Table 5).

### 3.4. Characteristics of Subjects without Ultrasonographic Fatty Liver Disease Stratified by Metabolic Syndrome

**Figure 3:**
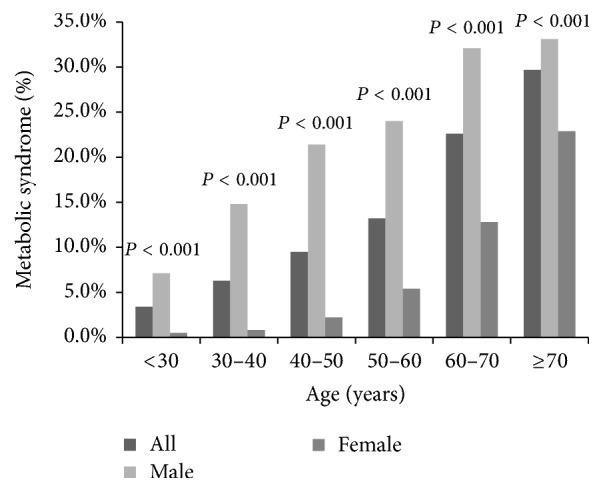
The prevalence of metabolic syndrome in subjects without sonographic fatty liver stratified by age and gender.

The demographics of subjects without ultrasonographic fatty liver disease are summarized in Table 6. The average age of the subjects was 50.9 years and 45% were male. Subjects with metabolic syndrome tended to be older in age, be male, have higher BMI, ALT, and GGT, and have lower platelet counts. The FLI and LAP were 37 and 47, respectively, in subjects with metabolic syndrome and 12 and 18, respectively, in subjects without metabolic syndrome. By multivariate analysis, older age, male sex, and higher BMI, ALT, and GGT were still associated with metabolic syndrome in subjects without fatty liver disease (Table 7). We further stratified subjects by age and sex, and the result revealed that male subjects had higher prevalence of metabolic syndrome, and the prevalence of metabolic syndrome increased with age (Figure 3).

### 3.5. Validation of the FLI and LAP for Identifying Metabolic Syndrome in Subjects without Ultrasonographic Fatty Liver Disease

The predictive ability of FLI and LAP to identify metabolic syndrome was determined by comparing their AUROC curve values (Table 8). The AUROC curve values of the LAP and FLI to predict the presence of metabolic syndrome were 0.871 and 0.879, respectively. After the subjects were stratified by sex, the AUROC curve values of the LAP and FLI were 0.921 and 0.909, respectively, in women and 0.844 and 0.814, respectively, in men.

## 4. Discussion

The FLI and LAP are indexes originally designed to assess the risk of fatty liver and cardiovascular disease, respectively, and both have been shown to be good markers of metabolic syndrome [4, 10, 12, 13]. In the present study, fatty liver disease was closely associated with metabolic syndrome, and both the FLI and LAP were predictive of metabolic syndrome. For people without fatty liver, both indexes were still strong predictors of metabolic syndrome.

Twenty-seven percent of the population aged more than 25 years in the US [22] and approximately 12% between 1999 and 2002 in Taiwan have been reported to have metabolic syndrome [23]. However, the prevalence of metabolic syndrome was much higher in the present study (28%), which may be because of the westernization of diet, greater awareness of the syndrome, or higher socioeconomic status of the subjects. It was noteworthy that the proportion of subjects with metabolic syndrome increased with age and the trend existed in both sexes (Figure 2(b)).

The prevalence of NAFLD, which varies by the diagnostic modality and ethnicity, ranges from 23% to 51% in Asian populations, and the prevalence of fatty liver disease in this study population falls within this range at 44.5% [24, 25]. Fatty liver disease was the strongest factor associated with metabolic syndrome in both sexes (Table 2 and Figure 2(a)). However, metabolic syndrome also exists in subjects without fatty liver disease, a group of subjects who have cardiovascular risk but is rarely focused on. Han et al. observed that 12% of subjects without ultrasonographic fatty liver disease at the end of the study developed metabolic syndrome in South Korea [26]. Up to 26.5% of subjects with mild or absent liver steatosis were also noted to have metabolic syndrome in Italy [27]. Metabolic syndrome can even occur in children without fatty liver disease. Schwimmer et al. defined the absence of NAFLD as the combination of a normal ALT level (<30 U/L) and the absence of hepatomegaly and found that metabolic syndrome exists in 15% of children with a mean age of 12.7 years without NAFLD [28]. After subjects with ultrasonographic fatty liver disease were excluded, the prevalence of metabolic syndrome in our study fell to 13.7%, similar to that in previous studies. Besides, an age-related increasing trend in the prevalence of metabolic syndrome was observed in both subjects with and without fatty liver disease (Figure 3).

Prediabetes has been recognized as a cardiometabolic risk factor and its phenotypes have been thoroughly assessed [29]. In this study, we investigated the role of the FLI and LAP to predict IFG after the subjects were stratified by the diagnosis of fatty liver disease and BMI. Interestingly, the results showed that the FLI and LAP had better predictive abilities for lean subjects and those without fatty liver than their counterparts. These findings suggest that the FLI and LAP could help clinical physicians identify a high-risk group of cardiometabolic diseases in these two commonly overlooked populations.

Bedogni et al. developed the FLI as an accurate index which correlates well with ultrasonographic fatty liver disease. The FLI has limited utility for the quantification of hepatic steatosis [30–32], but it has been validated by abdominal ultrasonography in several populations with an AUROC curve between 0.930 and 0.840 in Western countries to identify fatty liver disease, though the accuracy is less prominent in Asian countries probably because of variation of ethnicity, dietary, and environmental factors [4, 33].

Furthermore, the FLI is associated with cardiovascular risk factors including hypertension and carotid plaques [9, 34] and can predict cardiovascular and liver-related mortality [35–37]. As cardiovascular disease, obesity, NAFLD, and metabolic syndrome are intertwined, Rogulj et al. suggested that the FLI may be an optimal diagnostic method for metabolic syndrome in terms of sensitivity and specificity [10]. Their findings were comparable with those of the present study in that the FLI was better than other variables to predict metabolic syndrome with an AUROC curve of 0.875. We further analyzed the performance of the FLI to identify metabolic syndrome in subjects without fatty liver disease. Our results showed that the FLI was a reliable tool to predict metabolic syndrome with an AUROC curve of 0.879. Accordingly, subjects without fatty liver disease and a high FLI may also need an intensified counselling plan.

The LAP, which includes TG concentration and WC, was first proposed by Kahn to recognize cardiovascular risk [14, 38]. The LAP was further noticed to have a strong association with insulin resistance [39, 40], glucose dysregulation [40, 41], and type 2 DM [42, 43] and was also associated with the stroke incidence in a 9.2-year prospective Chinese study [44]. Bedogni et al. concluded that the LAP can be a good marker of liver steatosis and the conclusion was validated in Korea although the accuracy is lower in the Asian population [12, 45]. Several studies found that the LAP could be a good indicator of metabolic syndrome with an AUROC curve greater than 0.9 [13, 46–48]. The present study revealed that the LAP was a useful tool to identify subjects with metabolic syndrome, which was comparable to the findings of previous studies. However, in our analysis, the LAP was found to be a better predictor of metabolic syndrome among women, which was different from the results of another Taiwanese study which found that the LAP was better to recognize metabolic syndrome among men [47]. The inclusion of people aged over 50 years and the small sample size may explain the difference in the results. Furthermore, we also found that the LAP could reliably identify metabolic syndrome (AUROC: 0.879) even in subjects without fatty liver, and the predictive power was better among women as well.

TG values already have a high power to predict metabolic syndrome. With the addition of the FLI and LAP, more people with risk of cardiovascular disease can be recognized. The awareness of the risk with further implementation of an intensive counselling plan will be of great importance to prevent cardiovascular disease.

The large sample size and detailed biochemistry data are the strengths of this study. However, there are several noteworthy limitations. First, the study population had a higher socioeconomic status and subjects could afford the expense of a physical check-up, so the results might not represent the general population. Second, alcohol consumption was not evaluated in the present study. However, the prevalence of alcoholism was surveyed to be 1.5% in Taiwanese communities [49], and the impact on our results should be small. Third, liver biopsy is the gold standard for the diagnosis of fatty liver disease. However, the invasiveness of that procedure is not justified for surveillance in the general population. The diagnosis of fatty liver disease was made by at least two of three abnormal findings on abdominal ultrasonography: diffusely increased echogenic liver as compared with the kidney or spleen, vascular blurring, and deep attenuation of the ultrasound signal with sensitivity of 89% and specificity of 93% [50, 51]. Fourth, we could not analyze impaired glucose tolerance, another important phenotype of cardiometabolic risk, because the oral glucose tolerance test was not performed in our cohort. Further studies are warranted to elucidate the correlation between the FLI/LAP and cardiometabolic risk.

In conclusion, metabolic syndrome increases risk of cardiovascular disease, and the FLI and LAP could be used to recognize the syndrome in people without fatty liver disease who also require lifestyle modifications and counseling in addition to their counterparts with fatty liver disease.

## Figures and Tables

**Table 1:** Characteristics of subjects with and without metabolic syndrome.

	All	With metabolic syndrome	Without metabolic syndrome	*P* value
	(*n* = 29,797)	(*n* = 8564)	(*n* = 21,233)	
BMI, kg/m^2^^*∗*^	23.81 ± 3.58	26.23 ± 3.31	22.84 ± 3.21	<0.001
Age, years^*∗*^	52.2 ± 13.3	56.3 ± 12.5	50.6 ± 13.2	<0.001
Sex (M/F) (%)	16,098/13,699	6525/2039	9573/11,660	<0.001
	(54.0/46.0)	(76.2/23.8)	(45.1/54.9)	
WC, cm^*∗*^	83.8 ± 10.3	91.5 ± 8.4	80.7 ± 9.3	<0.001
SBP, mmHg^*∗*^	124.3 ± 18.6	134.9 ± 17.2	120.0 ± 17.4	<0.001
DBP, mmHg^*∗*^	77.5 ± 14.3	83.7 ± 17.5	75.0 ± 11.8	<0.001
Fasting glucose, mg/dL^*∗*^	95.5 ± 24.8	110.4 ± 35.3	89.5 ± 15.3	<0.001
Cholesterol, mg/dL^*∗*^	199.2 ± 37.0	203.1 ± 38.1	197.6 ± 36.5	<0.001
HDL, mg/dL^*∗*^	53.7 ± 15.0	42.9 ± 10.12	58.0 ± 14.5	<0.001
LDL, mg/dL^*∗*^	125.3 ± 32.9	129.0 ± 33.3	123.8 ± 32.6	<0.001
TG, mg/dL^*∗*^	130.4 ± 88.1	201.3 ± 111.5	101.8 ± 55.1	<0.001
AST, IU/L^*∗*^	23.1 ± 13.2	26.0 ± 18.2	21.9 ± 10.3	<0.001
ALT, IU/L^*∗*^	27.0 ± 22.2	35.1 ± 29.1	23.8 ± 17.6	<0.001
GGT, IU/L^*∗*^	24.8 ± 36.8	34.2 ± 50.9	21.0 ± 28.4	<0.001
Platelet, 1000/mm^3^^*∗*^	249.8 ± 60.3	247.5 ± 62.0	250.8 ± 59.6	<0.001
Fatty liver (yes/no) (%)	13,255/16,542	6295/2269	6960/14,273	<0.001
	(44.5/55.5)	(73.5/26.5)	(32.8/67.2)	
FLI	27.24 ± 24.18	50.22 ± 22.81	17.97 ± 17.65	<0.001
LAP	35.28 ± 33.59	64.22 ± 43.52	23.61 ± 18.57	<0.001

^*∗*^Expressed as mean ± standard deviation.

BMI, body mass index; M, male; F, female; WC, waist circumference; SBP, systolic blood pressure; DBP, diastolic blood pressure; HDL, high-density lipoprotein; LDL, low-density lipoprotein; TG, triglyceride; AST, aspartate aminotransferase; ALT, alanine aminotransferase; GGT, gamma-glutamyl transferase; FLI, fatty liver index; LAP, lipid accumulation product.

**Table 2:** Factors associated with metabolic syndrome by multivariate analysis.

	Odds ratio	95% confidence interval	*P* value
*All subjects*			
BMI	1.272	1.258–1.285	<0.001
Age	1.041	1.038–1.043	<0.001
ALT	1.006	1.005–1.008	<0.001
GGT	1.005	1.004–1.006	<0.001
Platelet	1.002	1.001–1.002	<0.001
Fatty liver	2.499	2.339–2.670	<0.001
Male gender	3.005	2.811–3.214	<0.001
*Females*			
BMI	1.311	1.288–1.335	<0.001
Age	1.065	1.059–1.071	<0.001
ALT	1.007	1.004–1.009	<0.001
GGT	1.004	1.002–1.005	<0.001
Platelet	1.001	1.001–1.002	0.002
Fatty liver	3.275	2.888–3.714	<0.001
*Males*			
BMI	1.242	1.225–1.259	<0.001
Age	1.032	1.029–1.035	<0.001
ALT	1.006	1.004–1.008	<0.001
GGT	1.005	1.004–1.007	<0.001
Platelet	1.002	1.001–1.002	<0.001
Fatty liver	2.205	2.040–2.384	<0.001

BMI, body mass index; ALT, alanine aminotransferase; GGT, gamma-glutamyl transferase.

**Table 3:** Comparison of AUROC curve values among noninvasive markers for predicting metabolic syndrome.

	AUROC	95% confidence interval	Standard error	*P* value
*All subjects*				
LAP	0.884	0.893–0.901	0.002	<0.001
FLI	0.875	0.871–0.879	0.002	<0.001
TG	0.853	0.849–0.858	0.002	<0.001
HDL	0.820	0.815–0.825	0.003	<0.001
WC	0.817	0.812–0.821	0.002	<0.001
BMI	0.785	0.779–0.790	0.003	<0.001
Fasting glucose	0.775	0.769–0.781	0.003	<0.001
GGT	0.724	0.717–0.730	0.003	<0.001
Fatty liver	0.704	0.697–0.710	0.003	<0.001
ALT	0.691	0.684–0.698	0.003	<0.001
LDL	0.548	0.541–0.556	0.004	<0.001
Cholesterol	0.543	0.536–0.550	0.004	<0.001
*Female subjects*				
LAP	0.927	0.922–0.933	0.003	<0.001
FLI	0.916	0.910–0.922	0.003	<0.001
TG	0.874	0.866–0.882	0.004	<0.001
WC	0.853	0.845–0.862	0.004	<0.001
Fasting glucose	0.850	0.840–0.860	0.005	<0.001
BMI	0.833	0.824–0.842	0.005	<0.001
HDL	0.825	0.816–0.835	0.005	<0.001
Fatty liver	0.747	0.736–0.759	0.006	<0.001
GGT	0.733	0.721–0.744	0.006	<0.001
ALT	0.693	0.681–0.706	0.006	<0.001
LDL	0.591	0.577–0.604	0.007	<0.001
Cholesterol	0.575	0.562–0.589	0.007	<0.001
*Male subjects*				
LAP	0.856	0.850–0.862	0.003	<0.001
TG	0.825	0.818–0.831	0.003	<0.001
FLI	0.818	0.812–0.825	0.003	<0.001
HDL	0.780	0.773–0.788	0.004	<0.001
Fasting glucose	0.744	0.736–0.752	0.004	<0.001
WC	0.741	0.733–0.748	0.004	<0.001
BMI	0.723	0.716–0.731	0.004	<0.001
GGT	0.659	0.650–0.667	0.004	<0.001
ALT	0.634	0.625–0.643	0.004	<0.001
Fatty liver	0.658	0.650–0.667	0.004	<0.001
Cholesterol	0.542	0.533–0.551	0.005	<0.001
LDL	0.518	0.508–0.527	0.005	<0.001

AUROC, area under the receiver operating characteristic; LAP, lipid accumulation product; FLI, fatty liver index; TG, triglyceride; HDL, high-density lipoprotein; WC, waist circumference; BMI, body mass index; GGT, gamma-glutamyl transferase; ALT: alanine aminotransferase; LDL, low-density lipoprotein.

**Table 4:** Characteristics of subjects with IFG.

	IFG	Non-IFG	*P* value
	(*n* = 5001)	(*n* = 22,970)	
BMI, kg/m^2^^*∗*^	25.28 ± 3.48	23.34 ± 3.44	<0.001
Age, years^*∗*^	57.7 ± 11.8	50.3 ± 13.1	<0.001
Sex (M/F) (%)	3036/1965	11,859/11,111	<0.001
	(60.7/39.3)	(51.6/48.4)	
WC, cm^*∗*^	88.2 ± 9.4	82.3 ± 9.9	<0.001
SBP, mmHg^*∗*^	131.8 ± 18.5	121.7 ± 17.8	<0.001
DBP, mmHg^*∗*^	81.2 ± 15.5	76.4 ± 13.3	<0.001
Fasting glucose, mg/dL^*∗*^	107.6 ± 6.8	87 ± 7.6	<0.001
Cholesterol, mg/dL^*∗*^	203.8 ± 37.6	197.9 ± 36.5	<0.001
HDL, mg/dL^*∗*^	50.4 ± 13.4	55 ± 15.3	<0.001
LDL, mg/dL^*∗*^	129 ± 32.7	124.4 ± 32.7	<0.001
TG, mg/dL^*∗*^	152.3 ± 90	120.9 ± 80.8	<0.001
AST, IU/L^*∗*^	25.2 ± 19	22.3 ± 10.5	<0.001
ALT, IU/L^*∗*^	31.6 ± 28.9	25.4 ± 19.2	<0.001
GGT, IU/L^*∗*^	30.7 ± 51.4	22.4 ± 27.3	<0.001
Platelet, 1000/mm^3^^*∗*^	245.3 ± 60.1	251.4 ± 59.9	<0.001
Fatty liver (yes/no) (%)	3150/1851	8737/14,233	<0.001
	(63.0/37.0)	(38.0/62.0)	
FLI	38.15 ± 25.02	23.3 ± 22.28	<0.001
LAP	46.51 ± 35.15	30.69 ± 29.6	<0.001

^*∗*^Expressed as mean ± standard deviation.

IFG, impaired fasting glucose; BMI, body mass index; M, male; F, female; WC, waist circumference; SBP, systolic blood pressure; DBP, diastolic blood pressure; HDL, high-density lipoprotein; LDL, low-density lipoprotein; TG, triglyceride; AST, aspartate aminotransferase; ALT, alanine aminotransferase; GGT, gamma-glutamyl transferase; LAP, lipid accumulation product.

**Table 5:** Comparison of AUROC curve values among noninvasive markers for predicting IFG.

	AUROC	95% confidence interval	Standard error	*P* value
*All*				
FLI	0.689	0.004	0.682–0.697	<0.001
LAP	0.675	0.004	0.667–0.683	<0.001
*With fatty liver*				
FLI	0.609	0.006	0.598–0.62	<0.001
LAP	0.602	0.006	0.591–0.613	<0.001
*Without fatty liver*				
FLI	0.669	0.006	0.656–0.681	<0.001
LAP	0.643	0.007	0.629–0.656	<0.001
*BMI (lean and normal)*				
FLI	0.673	0.008	0.658–0.688	<0.001
LAP	0.642	0.008	0.626–0.659	<0.001
*BMI (overweight and obesity)*				
FLI	0.616	0.005	0.606–0.627	<0.001
LAP	0.605	0.005	0.595–0.615	<0.001

IFG, impaired fasting glucose; AUROC, area under the receiver operating characteristic; FLI, fatty liver index; LAP, lipid accumulation product; BMI, body mass index.

**Table 6:** Characteristics of non-fatty liver subjects with and without metabolic syndrome.

	All	With metabolic syndrome	Without metabolic syndrome	*P* value
	(*n* = 16,542)	(*n* = 2269)	(*n* = 14,273)	
BMI, kg/m^2^^*∗*^	22.32 ± 2.90	24.79 ± 2.72	21.93 ± 2.73	<0.001
Age, years^*∗*^	50.9 ± 14.1	58.6 ± 13.8	49.6 ± 13.8	<0.001
Sex (M/F) (%)	7388/9154	1765/504	5623/8650	<0.001
	(44.7/55.3)	(77.8/22.2)	(39.4/60.6)	
WC, cm^*∗*^	79.5 ± 8.9	88.4 ± 7.3	78.1 ± 8.4	<0.001
SBP, mmHg^*∗*^	121.1 ± 18.5	136.0 ± 17.6	118.8 ± 17.6	<0.001
DBP, mmHg^*∗*^	75.3 ± 11.6	82.6 ± 11.1	74.1 ± 11.3	<0.001
Fasting glucose, mg/dL^*∗*^	90.7 ± 19.1	107.1 ± 32.7	88.1 ± 14.2	<0.001
Cholesterol, mg/dL^*∗*^	194.5 ± 36.0	195.8 ± 37.7	194.3 ± 35.7	0.058
HDL, mg/dL^*∗*^	58.2 ± 15.5	44.2 ± 11.4	60.4 ± 14.9	<0.001
LDL, mg/dL^*∗*	120.5 ± 31.7	124.2 ± 32.3	119.9 ± 31.6	<0.001
TG, mg/dL^*∗*^	101.0 ± 57.2	168.0 ± 81.5	90.3 ± 43.7	<0.001
AST, IU/L^*∗*^	21.1 ± 11.4	23.1 ± 23.9	20.8 ± 7.7	<0.001
ALT, IU/L^*∗*^	21.2 ± 17.2	26.2 ± 33.2	20.4 ± 12.7	<0.001
GGT, IU/L^*∗*^	20.0 ± 33.9	30.4 ± 66.9	18.3 ± 24.5	<0.001
Platelet, 1000/mm^3^^*∗*^	247.6 ± 60.9	238.7 ± 65.5	249.0 ± 60.0	<0.001
FLI	15.61 ± 16.27	36.99 ± 20.27	12.21 ± 12.54	<0.001
LAP	22.35 ± 18.81	47.10 ± 27.58	18.41 ± 13.27	<0.001

^*∗*^Expressed as mean ± standard deviation.

BMI, body mass index; M, male; F, female; WC, waist circumference; SBP, systolic blood pressure; DBP, diastolic blood pressure; HDL, high-density lipoprotein; LDL, low-density lipoprotein; TG, triglyceride; AST, aspartate aminotransferase; ALT, alanine aminotransferase; GGT, gamma-glutamyl transferase; FLI, fatty liver index; LAP, lipid accumulation product.

**Table 7:** Risk factors of metabolic syndrome in subjects with non-fatty liver disease by multivariate analysis.

	Odds ratio	95% confidence interval	*P* value
*All subjects*			
BMI	1.371	1.346–1.397	<0.001
Age	1.042	1.038–1.046	<0.001
ALT	1.007	1.004–1.010	<0.001
GGT	1.004	1.002–1.006	<0.001
Platelet	1.001	1.001–1.002	0.001
Male gender	4.071	3.628–4.568	<0.001
*Female subjects*			
BMI	1.382	1.339–1.426	<0.001
Age	1.080	1.070–1.089	<0.001
ALT	1.010	1.005–1.015	<0.001
Platelet	1.002	1.000–1.003	0.028
*Male subjects*			
BMI	1.351	1.320–1.383	<0.001
Age	1.032	1.028–1.037	<0.001
ALT	1.005	1.001–1.009	0.021
GGT	1.005	1.003–1.007	<0.001
Platelet	1.002	1.001–1.003	0.002

BMI, body mass index; ALT, alanine aminotransferase; GGT, gamma-glutamyl transferase.

**Table 8:** Comparison of AUROC curve values among non-fatty liver subjects with metabolic syndrome.

	AUROC	95% confidence interval	Standard error	*P* value
*All subjects*				
FLI	0.879	0.872–0.885	0.003	<0.001
LAP	0.871	0.863–0.879	0.004	<0.001
Triglyceride	0.828	0.818–0.838	0.005	<0.001
WC	0.827	0.819–0.835	0.004	<0.001
HDL	0.822	0.832–0.813	0.005	<0.001
BMI	0.783	0.774–0.792	0.005	<0.001
Fasting glucose	0.774	0.762–0.785	0.006	<0.001
GGT	0.693	0.682–0.704	0.006	<0.001
Age	0.679	0.667–0.691	0.006	<0.001
ALT	0.619	0.607–0.631	0.006	<0.001
LDL	0.543	0.531–0.556	0.006	<0.001
Cholesterol	0.513	0.501–0.526	0.007	0.04
*Female subjects*				
LAP	0.921	0.909–0.932	0.006	<0.001
FLI	0.909	0.898–0.921	0.006	<0.001
Triglyceride	0.860	0.842–0.878	0.009	<0.001
Fasting glucose	0.841	0.820–0.862	0.011	<0.001
WC	0.830	0.811–0.849	0.010	<0.001
HDL	0.822	0.841–0.803	0.010	<0.001
BMI	0.812	0.792–0.831	0.010	<0.001
Age	0.784	0.765–0.804	0.010	<0.001
GGT	0.673	0.650–0.697	0.012	<0.001
ALT	0.589	0.563–0.615	0.013	<0.001
LDL	0.574	0.548–0.600	0.013	<0.001
Cholesterol	0.547	0.520–0.574	0.014	<0.001
*Male subjects*				
LAP	0.844	0.834–0.855	0.005	<0.001
FLI	0.814	0.803–0.825	0.005	<0.001
Triglyceride	0.787	0.774–0.801	0.007	<0.001
HDL	0.772	0.786–0.759	0.007	<0.001
WC	0.757	0.745–0.768	0.006	<0.001
Fasting glucose	0.738	0.724–0.753	0.007	<0.001
BMI	0.723	0.711–0.736	0.006	<0.001
Age	0.615	0.601–0.630	0.007	<0.001
GGT	0.615	0.600–0.630	0.008	<0.001
ALT	0.559	0.544–0.575	0.008	<0.001
Cholesterol	0.517	0.502–0.533	0.008	0.029
LDL	0.514	0.499–0.529	0.008	0.074

AUROC, area under the receiver operating characteristic; FLI, fatty liver index; LAP, lipid accumulation product; TG, triglyceride; WC, waist circumference; HDL, high-density lipoprotein; BMI, body mass index; GGT, gamma-glutamyl transferase; ALT, alanine aminotransferase; LDL, low-density lipoprotein.
